# Poly[bis­(dimethyl­ammonium) [bis­(dimethyl­amine-κ*N*)tris­(μ_2_-terephthalato-κ^2^ *O* ^1^:*O* ^4^)dizinc(II)] *N*,*N*-dimethyl­formamide disolvate hexa­hydrate]

**DOI:** 10.1107/S1600536809030177

**Published:** 2009-08-15

**Authors:** Jie Xiao, Hu Zhou, Ai-Hua Yuan

**Affiliations:** a School of Materials Science and Engineering, Jiangsu University of Science and Technology, Zhenjiang 212003, People’s Republic of China

## Abstract

The title compound, {(C_2_H_8_N)_2_[Zn_2_(C_8_H_4_O_4_)_3_(C_2_H_7_N)_2_]·2C_3_H_7_NO·6H_2_O}_*n*_, consists of two-dimensional non-inter­penetrated sheets with 6^3^ topology, which are stacked together in an …*ABAB*… packing mode along the *c* axis. The distance between adjacent *A* and *B* sheets is *ca* 7.3 Å. In the structure, the Zn^II^ center is coordinated by three O atoms from three terephthalate groups and one N atom from one dimethyl­amine ligand, adopting a distorted tetra­hedral geometry. All solvent water mol­ecules are disordered. In the structure, N—H⋯O and O—H⋯O hydrogen bonds are observed.

## Related literature

For background to metal-organic frameworks, see: Kitagawa *et al.* (2004 ▶); Rowsell *et al.* (2004 ▶); Tranchemontagne *et al.* (2008 ▶); Wang *et al.* (2008 ▶); Hawxwell *et al.* (2006 ▶). For related structures, see: Wang *et al.* (2007 ▶); Go *et al.*(2007 ▶); Dai *et al.* (2004 ▶); Guo *et al.* (2009 ▶); He *et al.* (2005 ▶); Zhu *et al.* (2007 ▶); Clausen *et al.* (2005 ▶); Dybtsev *et al.* (2004 ▶); Robin & Fromm (2006 ▶); Rowsell & Yaghi (2004 ▶); Suh *et al.* (2008 ▶); Wu *et al.* (2005 ▶).
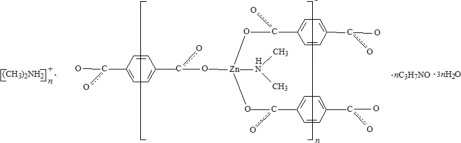


## Experimental

### 

#### Crystal data

(C_2_H_8_N)_2_[Zn_2_(C_8_H_4_O_4_)_3_(C_2_H_7_N)_2_]·2C_3_H_7_NO·6H_2_O
*M* *_r_* = 1059.72
Orthorhombic, 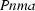

*a* = 18.421 (6) Å
*b* = 30.906 (11) Å
*c* = 11.346 (4) Å
*V* = 6459 (4) Å^3^
*Z* = 4
Mo *K*α radiation
μ = 0.80 mm^−1^
*T* = 291 K
0.28 × 0.22 × 0.20 mm

#### Data collection

Bruker SMART APEX CCD diffractometer
Absorption correction: multi-scan (*SADABS*; Bruker, 2004 ▶) *T*_min_ = 0.81, *T*_max_ = 0.85
49068 measured reflections
6463 independent reflections
3985 reflections with *I* > 2σ(*I*)
*R*_int_ = 0.090

#### Refinement

*R*[*F* ^2^ > 2σ(*F* ^2^)] = 0.049
*wR*(*F* ^2^) = 0.106
*S* = 1.04
6463 reflections
358 parameters
H-atom parameters constrained
Δρ_max_ = 0.42 e Å^−3^
Δρ_min_ = −0.44 e Å^−3^

### 

Data collection: *SMART* (Bruker, 2004 ▶); cell refinement: *SAINT* (Bruker, 2004 ▶); data reduction: *SAINT*; program(s) used to solve structure: *SHELXTL* (Sheldrick, 2008 ▶); program(s) used to refine structure: *SHELXTL*; molecular graphics: *SHELXTL*; software used to prepare material for publication: *SHELXTL*.

## Supplementary Material

Crystal structure: contains datablocks I, global. DOI: 10.1107/S1600536809030177/at2837sup1.cif

Structure factors: contains datablocks I. DOI: 10.1107/S1600536809030177/at2837Isup2.hkl

Additional supplementary materials:  crystallographic information; 3D view; checkCIF report

## Figures and Tables

**Table 1:** Hydrogen-bond geometry (Å, °)

*D*—H⋯*A*	*D*—H	H⋯*A*	*D*⋯*A*	*D*—H⋯*A*
N1—H1*A*⋯O4^i^	0.91	2.27	3.109 (3)	152
N1—H1*A*⋯O2	0.91	2.54	3.040 (3)	115
N2—H2*A*⋯O4^ii^	0.91	1.93	2.770 (3)	153
N2—H2*B*⋯O15	0.89	2.64	3.241 (7)	127
O8—H8*X*⋯O12^iii^	0.85	2.09	2.519 (9)	111
O8—H8*X*⋯N3^ii^	0.85	2.59	3.329 (5)	147
O9—H9*X*⋯O9^iv^	0.85	1.61	2.063 (11)	110
O10—H10*X*⋯O7	0.85	1.95	2.524 (7)	124
O11—H11*Y*⋯O9	0.85	2.47	2.927 (9)	115
O11—H11*X*⋯O15^iii^	0.85	1.73	2.552 (10)	161
O12—H12*X*⋯O8^v^	0.85	2.04	2.519 (9)	115
O13—H13*F*⋯O13^iv^	0.85	1.77	2.460 (14)	137
O13—H13*F*⋯O14	0.85	2.08	2.650 (10)	124
O15—H15*X*⋯O11^v^	0.85	2.13	2.552 (10)	110
O16—H16*X*⋯O12	0.85	2.22	2.709 (9)	116
O16—H16*X*⋯O13	0.85	2.37	3.131 (9)	150
O16—H16*Y*⋯O10	0.85	2.62	3.079 (9)	116

Symmetry codes: (i) 
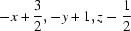
; (ii) 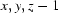; (iii) 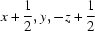; (iv) 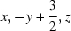; (v) 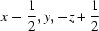.

**Fig. 1.**
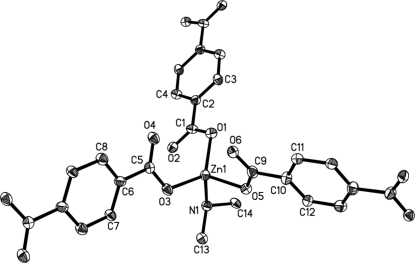
ORTEP diagram of the title compound. Displacement ellipsoids are drawn at the 30% probablity level. Hydrogen atoms, solvent dimethylamine, DMF, and water molecules are omitted for clarity.

**Fig. 2.**
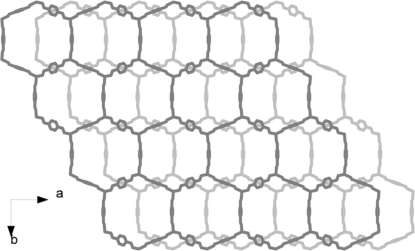
The stacking without interpenetration of sheets viewed from the c axis for the title compound.

**Fig. 3.**
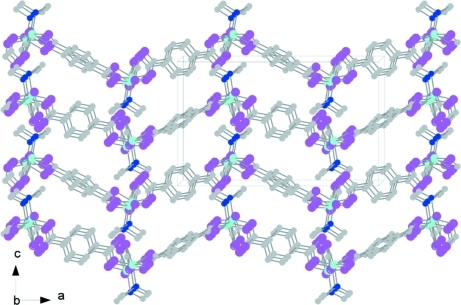
The two-dimensional corrugated sheets packing along the c axis of the title compound.

## supplementary crystallographic information

### Comment

The study of one-, two- or three-dimensional metal-organic frameworks (MOFs) has attracted much attention in the past decade due to not only their various intriguing framework topologies (Kitagawa *et al., *2004; Rowsell & Yaghi 2004; Robin *et al.*, 2006; Suh *et al.*, 2008) but also for their potential applications in gas storage (Rowsell *et al.*, 2004), separation (Dybtsev *et al.*, 2004) and catalysis (Wu *et al.*, 2005) *etc*. Particular attention has been attracted to the isolation and characterization of two-dimensional topologies that comprise just one kind of regular polygon based upon hexagons, squares and triangles corresponding to the 6^3^, 4^4^, 3^6^ topology, respectively. In the construction of hybrid frameworks, aromatic polycarboxylates, for example, terephalate (1,4-benzenedicarboxylate) are commonly used as bridging ligands (Wang *et al.*, 2008; Hawxwell *et al.*, 2006; Clausen *et al.*, 2005; Tranchemontagne *et al.*, 2008) because they can adopt monodentate or chelating coordination modes.

Here we employ terephalate as the bridging ligands to obtain the two-dimensional honeycomb networks (Go *et al.*, 2007; Wang *et al.*, 2007; He *et al.*, 2005) because of the availability of three-coordinated vertices. It is well known that metal-organic framework structures possessing large voids tend to form interpenetrated topologies. Some examples of honeycomb compounds which form interpenetrated networks have been reported (Dai *et al.*, 2004; Guo *et al.*, 2009). In contrast, the title compound is an unusual example of two-dimensional noninterpenetrated sheets with the 6^3^ topology.

The Zn center is coordinated by three O atoms from three terephalate groups and one N atom from dimethylamine ligand, adopting a tetrahedral geometry (Fig. 1). The bond lengths of Zn—O range from 1.956 Å to 1.984 Å, while the Zn—N bond distance is 2.063 Å (Table 1). The Zn centers are linked by terephalate ligands, resulting in two-dimensional corrugated sheets stacking along the *c* axis (Fig. 2 and 3). These two-dimensional sheets are stacked together in an ABAB packing mode along the *c* axis. The distance between the adjacent A and B sheets is *ca* 7.3 Å (Zn···Zn distance). The offset distance between the adjacent sheets is *ca*. 12.3 Å along the *a* axis in the *ab* plane. Under hydrothermal conditions, it is worthy to note that the DMF solvent is decomposed into dimethylamine, which coordinates to Zn center in the structure. The similar examples can be found in other metal-organic frameworks (Zhu *et al.*, 2007). In the structure, it is observed N—H···O and O—H···O hydrogen bonds (Table 2).

### Experimental

A mixture of Zn(NO_3_)_2_.6H_2_O (29.7 mg, 0.1 mmol) and terephthalic acid (16.6 mg, 0.1 mmol) in a molar ratio of 1:1 combined with 6 ml DMF was stirred for 20 min at room temperature. Then the solution was heated hydrothermally in a 25 ml Teflon-lined stainless-steel vessel at 443 K for three days under autogenous pressure. Slow cooling of the resulting solution to room temperature at the rate of 10 °C.h^-1^ afforded colourless block crystals suitable for single-crystal X-ray structure analysis. Yield: 27%. These crystals were separated, washed thoroughly with DMF, and dried. Analysis calculated for C_19_H_33_N_3_O_10_Zn: C 43.15; H 6.29; N 7.95%. Found: C 43.12; H 6.26; N 7.99%.

### Refinement

The C(H) atoms of terephthalic acid ligands, dimethylamine ligands, DMF molecules and the N(H) atoms were all placed in calculated position (C—H = 0.93 Å or 0.96 Å, and N—H = 0.91 Å) and refined using a riding model, with *U*_ĩso_(H) = 1.2*U*_eq_(C, N) or *U*_ĩso_(H) = 1.5*U*_eq_(C). All solvent water molecules are disordered, and the O(H) atoms were located in a difference Fourier map and refined as riding (O—H = 0.85 Å), with *U*_ĩso_(H) = 1.2 or 1.5*U*_eq_(O).

### Crystal data

**Table:**

(C_2_H_8_N)_2_[Zn_2_(C_8_H_4_O_4_)_3_(C_2_H_7_N)_2_]·2C_3_H_7_NO·6H_2_O	*F*(000) = 2232
*M**_r_* = 1059.72	*D*_x_ = 1.090 Mg m^−^^3^
Orthorhombic, *P**n**m**a*	Mo *K*α radiation, λ = 0.71073 Å
Hall symbol: -P 2ac 2n	Cell parameters from 6858 reflections
*a* = 18.421 (6) Å	θ = 2.2–23.6°
*b* = 30.906 (11) Å	µ = 0.80 mm^−^^1^
*c* = 11.346 (4) Å	*T* = 291 K
*V* = 6459 (4) Å^3^	Block, colourless
*Z* = 4	0.28 × 0.22 × 0.20 mm

### Data collection

**Table:**

Bruker SMART APEX CCD diffractometer	6463 independent reflections
Radiation source: sealed tube	3985 reflections with *I* > 2σ(*I*)
graphite	*R*_int_ = 0.090
φ and ω scans	θ_max_ = 26.0°, θ_min_ = 2.1°
Absorption correction: multi-scan (*SADABS*; Bruker, 2004)	*h* = −22→22
*T*_min_ = 0.81, *T*_max_ = 0.85	*k* = −35→38
49068 measured reflections	*l* = −13→13

### Refinement

**Table:**

Refinement on *F*^2^	Primary atom site location: structure-invariant direct methods
Least-squares matrix: full	Secondary atom site location: difference Fourier map
*R*[*F*^2^ > 2σ(*F*^2^)] = 0.049	Hydrogen site location: inferred from neighbouring sites
*wR*(*F*^2^) = 0.106	H-atom parameters constrained
*S* = 1.04	*w* = 1/[σ^2^(*F*_o_^2^) + (0.0437*P*)^2^] where *P* = (*F*_o_^2^ + 2*F*_c_^2^)/3
6463 reflections	(Δ/σ)_max_ < 0.001
358 parameters	Δρ_max_ = 0.42 e Å^−^^3^
0 restraints	Δρ_min_ = −0.44 e Å^−^^3^

### Special details

**Table:**

Geometry. All esds (except the esd in the dihedral angle between two l.s. planes) are estimated using the full covariance matrix. The cell esds are taken into account individually in the estimation of esds in distances, angles and torsion angles; correlations between esds in cell parameters are only used when they are defined by crystal symmetry. An approximate (isotropic) treatment of cell esds is used for estimating esds involving l.s. planes.
Refinement. Refinement of F^2^ against ALL reflections. The weighted R-factor wR and goodness of fit S are based on F^2^, conventional R-factors R are based on F, with F set to zero for negative F^2^. The threshold expression of F^2^ > 2sigma(F^2^) is used only for calculating R-factors(gt) etc. and is not relevant to the choice of reflections for refinement. R-factors based on F^2^ are statistically about twice as large as those based on F, and R- factors based on ALL data will be even larger.

### Fractional atomic coordinates and isotropic or equivalent isotropic displacement parameters (Å^2^)

**Table:**

	*x*	*y*	*z*	*U*_iso_*/*U*_eq_	Occ. (<1)
Zn1	0.748174 (18)	0.574369 (10)	0.82017 (3)	0.03184 (10)	
O1	0.65833 (10)	0.56316 (7)	0.90871 (16)	0.0365 (5)	
O2	0.66892 (11)	0.49777 (6)	0.82720 (15)	0.0346 (4)	
O3	0.84206 (11)	0.54729 (7)	0.85243 (16)	0.0379 (5)	
O4	0.80801 (10)	0.52970 (7)	1.03599 (16)	0.0396 (5)	
O5	0.75628 (10)	0.63783 (6)	0.79740 (15)	0.0330 (4)	
O6	0.78004 (10)	0.63646 (6)	0.98921 (16)	0.0346 (4)	
N1	0.73121 (13)	0.55959 (8)	0.6450 (2)	0.0358 (6)	
H1A	0.7200	0.5310	0.6400	0.043*	
C1	0.63617 (15)	0.52421 (10)	0.8892 (2)	0.0358 (7)	
C2	0.56500 (15)	0.51227 (9)	0.9466 (2)	0.0343 (6)	
C3	0.52584 (15)	0.54188 (9)	1.0140 (2)	0.0349 (6)	
H3	0.5431	0.5699	1.0241	0.042*	
C4	0.53859 (14)	0.47065 (9)	0.9343 (2)	0.0338 (6)	
H4	0.5648	0.4507	0.8902	0.041*	
C5	0.85371 (15)	0.53206 (9)	0.9570 (2)	0.0303 (6)	
C6	0.92994 (15)	0.51533 (9)	0.9779 (2)	0.0348 (6)	
C7	0.97830 (16)	0.50915 (9)	0.8842 (2)	0.0347 (6)	
H7	0.9637	0.5152	0.8076	0.042*	
C8	0.95059 (16)	0.50634 (9)	1.0942 (2)	0.0375 (7)	
H8	0.9182	0.5105	1.1561	0.045*	
C9	0.76932 (16)	0.65657 (10)	0.8979 (3)	0.0390 (7)	
C10	0.76968 (16)	0.70493 (10)	0.8943 (3)	0.0388 (7)	
C11	0.77888 (16)	0.72718 (9)	0.9980 (2)	0.0368 (7)	
H11	0.7851	0.7121	1.0683	0.044*	
C12	0.75900 (15)	0.72744 (10)	0.7922 (3)	0.0399 (7)	
H12	0.7517	0.7125	0.7220	0.048*	
C13	0.79857 (15)	0.56728 (10)	0.5745 (2)	0.0361 (7)	
H13A	0.7985	0.5488	0.5065	0.054*	
H13B	0.8404	0.5610	0.6220	0.054*	
H13C	0.8001	0.5970	0.5497	0.054*	
C14	0.66929 (16)	0.58483 (10)	0.5948 (3)	0.0384 (7)	
H14A	0.6832	0.6146	0.5867	0.058*	
H14B	0.6282	0.5828	0.6466	0.058*	
H14C	0.6566	0.5733	0.5190	0.058*	
N2	0.71822 (14)	0.58391 (7)	0.1646 (2)	0.0354 (6)	
H2A	0.7433	0.5721	0.1039	0.043*	
H2B	0.6898	0.6051	0.1400	0.043*	
C15	0.76977 (15)	0.60535 (9)	0.2428 (3)	0.0367 (7)	
H15A	0.7630	0.5950	0.3219	0.055*	
H15B	0.7618	0.6360	0.2405	0.055*	
H15C	0.8184	0.5991	0.2175	0.055*	
C16	0.67670 (15)	0.54833 (9)	0.2260 (3)	0.0368 (7)	
H16A	0.7052	0.5371	0.2899	0.055*	
H16B	0.6666	0.5255	0.1709	0.055*	
H16C	0.6319	0.5597	0.2561	0.055*	
N3	0.99518 (13)	0.65769 (8)	0.7182 (2)	0.0429 (6)	
O7	0.94026 (11)	0.70680 (7)	0.62427 (17)	0.0433 (5)	
C17	1.07238 (16)	0.66164 (10)	0.6734 (3)	0.0437 (7)	
H17A	1.0783	0.6889	0.6338	0.066*	
H17B	1.0823	0.6385	0.6194	0.066*	
H17C	1.1055	0.6601	0.7387	0.066*	
C18	0.97423 (16)	0.61309 (10)	0.7612 (3)	0.0399 (7)	
H18A	0.9970	0.6076	0.8359	0.060*	
H18B	0.9899	0.5918	0.7051	0.060*	
H18C	0.9225	0.6115	0.7700	0.060*	
C19	0.94719 (16)	0.69498 (10)	0.7315 (3)	0.0422 (8)	
H19	0.9272	0.7069	0.7995	0.051*	
O8	0.9438 (3)	0.67157 (17)	−0.0031 (4)	0.0440 (13)	0.40
H8X	0.9746	0.6689	−0.0584	0.053*	0.40
H8Y	0.9637	0.6652	0.0622	0.053*	0.40
O9	0.8541 (3)	0.71662 (18)	0.2652 (5)	0.0474 (13)	0.40
H9X	0.8835	0.7354	0.2384	0.057*	0.40
H9Y	0.8109	0.7258	0.2577	0.057*	0.40
O10	0.8853 (4)	0.6581 (2)	0.4696 (6)	0.0503 (18)	0.30
H10X	0.9200	0.6760	0.4780	0.060*	0.30
H10Y	0.8986	0.6333	0.4938	0.060*	0.30
O11	0.9212 (4)	0.6362 (2)	0.3470 (6)	0.0475 (18)	0.30
H11X	0.9660	0.6296	0.3475	0.057*	0.30
H11Y	0.9033	0.6426	0.2802	0.057*	0.30
O12	0.5802 (4)	0.6679 (2)	0.4913 (6)	0.0494 (18)	0.30
H12X	0.5468	0.6616	0.4425	0.059*	0.30
H12Y	0.5761	0.6937	0.5166	0.059*	0.30
O13	0.6421 (4)	0.7102 (2)	0.3095 (6)	0.0480 (18)	0.30
H13E	0.6165	0.7006	0.2528	0.072*	0.30
H13F	0.6247	0.7342	0.3331	0.072*	0.30
O14	0.5498 (4)	0.7708 (2)	0.2396 (6)	0.0467 (17)	0.30
H14E	0.5208	0.7614	0.1873	0.056*	0.30
H14F	0.5316	0.7931	0.2722	0.056*	0.30
O15	0.5532 (4)	0.6179 (2)	0.2001 (6)	0.0437 (17)	0.30
H15X	0.5213	0.6289	0.2452	0.052*	0.30
H15Y	0.5933	0.6311	0.2089	0.052*	0.30
O16	0.7248 (3)	0.67473 (17)	0.5303 (5)	0.0454 (13)	0.40
H16X	0.6995	0.6740	0.4677	0.055*	0.40
H16Y	0.7597	0.6925	0.5220	0.055*	0.40

### Atomic displacement parameters (Å^2^)

**Table:**

	*U*^11^	*U*^22^	*U*^33^	*U*^12^	*U*^13^	*U*^23^
Zn1	0.03285 (19)	0.02973 (17)	0.03293 (17)	0.00038 (15)	−0.00052 (13)	−0.00070 (13)
O1	0.0329 (11)	0.0421 (12)	0.0345 (10)	−0.0038 (9)	−0.0018 (8)	0.0003 (9)
O2	0.0368 (11)	0.0372 (11)	0.0297 (9)	−0.0015 (9)	−0.0077 (8)	0.0008 (8)
O3	0.0346 (11)	0.0463 (12)	0.0327 (10)	0.0112 (10)	−0.0048 (8)	−0.0049 (9)
O4	0.0354 (12)	0.0464 (13)	0.0370 (10)	0.0076 (9)	−0.0040 (9)	−0.0066 (9)
O5	0.0330 (11)	0.0335 (10)	0.0326 (10)	−0.0033 (9)	−0.0025 (8)	−0.0010 (8)
O6	0.0371 (11)	0.0300 (10)	0.0367 (10)	−0.0031 (9)	−0.0023 (8)	−0.0054 (8)
N1	0.0339 (14)	0.0394 (14)	0.0340 (12)	0.0024 (10)	−0.0009 (9)	0.0002 (11)
C1	0.0351 (17)	0.0433 (18)	0.0291 (14)	−0.0044 (13)	0.0054 (12)	−0.0048 (13)
C2	0.0320 (16)	0.0354 (16)	0.0356 (14)	−0.0002 (13)	0.0018 (12)	0.0039 (12)
C3	0.0307 (16)	0.0355 (16)	0.0386 (14)	0.0028 (13)	−0.0045 (12)	−0.0043 (12)
C4	0.0273 (16)	0.0383 (16)	0.0357 (14)	−0.0078 (12)	−0.0012 (11)	−0.0057 (12)
C5	0.0290 (16)	0.0275 (14)	0.0342 (15)	−0.0017 (11)	−0.0044 (12)	−0.0034 (12)
C6	0.0333 (16)	0.0373 (16)	0.0339 (14)	−0.0013 (13)	0.0062 (12)	0.0002 (12)
C7	0.0338 (16)	0.0372 (16)	0.0330 (14)	0.0048 (13)	0.0015 (12)	−0.0067 (12)
C8	0.0285 (15)	0.0458 (18)	0.0381 (14)	0.0021 (13)	−0.0027 (12)	−0.0015 (13)
C9	0.0422 (18)	0.0308 (16)	0.0440 (16)	0.0016 (13)	0.0018 (13)	−0.0051 (14)
C10	0.0435 (18)	0.0312 (15)	0.0418 (15)	0.0020 (13)	−0.0039 (13)	0.0024 (13)
C11	0.0390 (16)	0.0367 (15)	0.0347 (15)	0.0005 (13)	0.0066 (12)	0.0017 (12)
C12	0.0430 (18)	0.0376 (16)	0.0393 (16)	0.0082 (14)	0.0040 (13)	0.0076 (12)
C13	0.0384 (17)	0.0369 (17)	0.0331 (14)	−0.0002 (13)	−0.0042 (12)	−0.0084 (12)
C14	0.0385 (17)	0.0413 (18)	0.0356 (15)	0.0044 (13)	0.0035 (13)	−0.0107 (12)
N2	0.0398 (14)	0.0327 (14)	0.0337 (12)	−0.0028 (11)	0.0066 (10)	0.0004 (10)
C15	0.0370 (17)	0.0334 (15)	0.0397 (15)	−0.0177 (13)	0.0012 (12)	−0.0102 (12)
C16	0.0316 (16)	0.0323 (16)	0.0465 (16)	−0.0011 (12)	0.0123 (13)	0.0159 (12)
N3	0.0420 (16)	0.0411 (15)	0.0455 (14)	0.0119 (12)	0.0147 (12)	0.0146 (11)
O7	0.0435 (13)	0.0435 (12)	0.0428 (11)	0.0153 (10)	0.0121 (9)	0.0114 (9)
C17	0.0383 (18)	0.0393 (18)	0.0534 (18)	−0.0045 (14)	−0.0091 (14)	−0.0137 (15)
C18	0.0336 (16)	0.0411 (17)	0.0451 (16)	−0.0150 (13)	−0.0167 (13)	0.0163 (14)
C19	0.0383 (18)	0.0489 (19)	0.0393 (16)	0.0136 (15)	0.0184 (13)	0.0106 (14)
O8	0.053 (3)	0.046 (3)	0.033 (2)	0.006 (3)	0.016 (2)	0.013 (2)
O9	0.036 (3)	0.059 (4)	0.047 (3)	−0.001 (3)	−0.004 (2)	−0.004 (3)
O10	0.050 (5)	0.050 (5)	0.050 (4)	−0.001 (4)	−0.019 (4)	0.007 (3)
O11	0.048 (4)	0.054 (5)	0.040 (4)	0.017 (4)	−0.014 (3)	−0.011 (3)
O12	0.050 (5)	0.054 (5)	0.044 (4)	−0.002 (4)	−0.002 (3)	−0.003 (3)
O13	0.054 (5)	0.039 (4)	0.051 (4)	−0.002 (3)	−0.003 (3)	−0.011 (3)
O14	0.041 (4)	0.055 (4)	0.044 (4)	−0.001 (3)	0.005 (3)	0.017 (3)
O15	0.038 (4)	0.042 (4)	0.051 (4)	0.020 (3)	0.006 (3)	0.017 (3)
O16	0.047 (3)	0.047 (3)	0.042 (3)	0.022 (3)	0.003 (2)	0.010 (2)

### Geometric parameters (Å, °)

**Table:**

Zn1—O3	1.956 (2)	N2—C16	1.509 (3)
Zn1—O1	1.967 (2)	N2—H2A	0.9063
Zn1—O5	1.984 (2)	N2—H2B	0.8851
Zn1—N1	2.063 (2)	C15—H15A	0.9600
O1—C1	1.290 (4)	C15—H15B	0.9600
O2—C1	1.236 (3)	C15—H15C	0.9600
O3—C5	1.295 (3)	C16—H16A	0.9600
O4—C5	1.231 (3)	C16—H16B	0.9600
O5—C9	1.301 (3)	C16—H16C	0.9600
O6—C9	1.224 (3)	N3—C19	1.460 (4)
N1—C14	1.494 (4)	N3—C18	1.512 (4)
N1—C13	1.495 (4)	N3—C17	1.515 (4)
N1—H1A	0.9100	O7—C19	1.277 (3)
C1—C2	1.510 (4)	C17—H17A	0.9600
C2—C4	1.382 (4)	C17—H17B	0.9600
C2—C3	1.394 (4)	C17—H17C	0.9600
C3—C4^i^	1.379 (4)	C18—H18A	0.9600
C3—H3	0.9300	C18—H18B	0.9600
C4—C3^i^	1.379 (4)	C18—H18C	0.9600
C4—H4	0.9300	C19—H19	0.9300
C5—C6	1.515 (4)	O8—H8X	0.8500
C6—C7	1.400 (4)	O8—H8Y	0.8501
C6—C8	1.402 (4)	O9—H9X	0.8500
C7—C8^ii^	1.416 (4)	O9—H9Y	0.8499
C7—H7	0.9300	O10—O11	1.682 (9)
C8—C7^ii^	1.416 (4)	O10—H10X	0.8500
C8—H8	0.9300	O10—H10Y	0.8501
C9—C10	1.495 (4)	O11—H11X	0.8501
C10—C12	1.366 (4)	O11—H11Y	0.8501
C10—C11	1.373 (4)	O12—H12X	0.8500
C11—C11^iii^	1.411 (6)	O12—H12Y	0.8499
C11—H11	0.9300	O13—H13E	0.8499
C12—C12^iii^	1.394 (6)	O13—H13F	0.8501
C12—H12	0.9300	O14—O14^iii^	1.287 (14)
C13—H13A	0.9600	O14—H14E	0.8501
C13—H13B	0.9600	O14—H14F	0.8501
C13—H13C	0.9600	O15—H15X	0.8499
C14—H14A	0.9600	O15—H15Y	0.8500
C14—H14B	0.9600	O16—H16X	0.8500
C14—H14C	0.9600	O16—H16Y	0.8499
N2—C15	1.459 (3)		
			
O3—Zn1—O1	124.97 (9)	N1—C14—H14A	109.5
O3—Zn1—O5	112.39 (8)	N1—C14—H14B	109.5
O1—Zn1—O5	107.69 (8)	H14A—C14—H14B	109.5
O3—Zn1—N1	102.68 (9)	N1—C14—H14C	109.5
O1—Zn1—N1	109.00 (9)	H14A—C14—H14C	109.5
O5—Zn1—N1	96.02 (9)	H14B—C14—H14C	109.5
C1—O1—Zn1	110.06 (17)	C15—N2—C16	112.4 (2)
C5—O3—Zn1	118.27 (18)	C15—N2—H2A	108.3
C9—O5—Zn1	109.86 (18)	C16—N2—H2A	108.4
C14—N1—C13	110.3 (2)	C15—N2—H2B	104.0
C14—N1—Zn1	111.51 (17)	C16—N2—H2B	112.7
C13—N1—Zn1	110.75 (17)	H2A—N2—H2B	111.0
C14—N1—H1A	108.1	N2—C15—H15A	109.5
C13—N1—H1A	108.1	N2—C15—H15B	109.5
Zn1—N1—H1A	108.1	H15A—C15—H15B	109.5
O2—C1—O1	124.1 (3)	N2—C15—H15C	109.5
O2—C1—C2	120.5 (3)	H15A—C15—H15C	109.5
O1—C1—C2	115.4 (2)	H15B—C15—H15C	109.5
C4—C2—C3	119.0 (3)	N2—C16—H16A	109.5
C4—C2—C1	119.3 (3)	N2—C16—H16B	109.5
C3—C2—C1	121.7 (3)	H16A—C16—H16B	109.5
C4^i^—C3—C2	119.6 (3)	N2—C16—H16C	109.5
C4^i^—C3—H3	120.2	H16A—C16—H16C	109.5
C2—C3—H3	120.2	H16B—C16—H16C	109.5
C3^i^—C4—C2	121.4 (3)	C19—N3—C18	122.1 (2)
C3^i^—C4—H4	119.3	C19—N3—C17	122.6 (2)
C2—C4—H4	119.3	C18—N3—C17	114.9 (2)
O4—C5—O3	125.1 (3)	N3—C17—H17A	109.5
O4—C5—C6	120.0 (2)	N3—C17—H17B	109.5
O3—C5—C6	114.9 (2)	H17A—C17—H17B	109.5
C7—C6—C8	121.0 (3)	N3—C17—H17C	109.5
C7—C6—C5	121.2 (2)	H17A—C17—H17C	109.5
C8—C6—C5	117.8 (2)	H17B—C17—H17C	109.5
C6—C7—C8^ii^	120.3 (3)	N3—C18—H18A	109.5
C6—C7—H7	119.9	N3—C18—H18B	109.5
C8^ii^—C7—H7	119.9	H18A—C18—H18B	109.5
C6—C8—C7^ii^	118.7 (3)	N3—C18—H18C	109.5
C6—C8—H8	120.6	H18A—C18—H18C	109.5
C7^ii^—C8—H8	120.6	H18B—C18—H18C	109.5
O6—C9—O5	123.1 (3)	O7—C19—N3	100.8 (2)
O6—C9—C10	122.0 (3)	O7—C19—H19	129.6
O5—C9—C10	115.0 (3)	N3—C19—H19	129.6
C12—C10—C11	119.3 (3)	H8X—O8—H8Y	109.5
C12—C10—C9	122.1 (3)	H9X—O9—H9Y	109.5
C11—C10—C9	118.6 (3)	O11—O10—H10X	93.4
C10—C11—C11^iii^	120.06 (17)	O11—O10—H10Y	78.0
C10—C11—H11	120.0	H10X—O10—H10Y	109.5
C11^iii^—C11—H11	120.0	O10—O11—H11X	118.2
C10—C12—C12^iii^	120.63 (19)	O10—O11—H11Y	119.4
C10—C12—H12	119.7	H11X—O11—H11Y	116.1
C12^iii^—C12—H12	119.7	H12X—O12—H12Y	111.8
N1—C13—H13A	109.5	H13E—O13—H13F	109.5
N1—C13—H13B	109.5	O14^iii^—O14—H14E	70.0
H13A—C13—H13B	109.5	O14^iii^—O14—H14F	144.0
N1—C13—H13C	109.5	H14E—O14—H14F	109.5
H13A—C13—H13C	109.5	H15X—O15—H15Y	109.8
H13B—C13—H13C	109.5	H16X—O16—H16Y	109.8
			
O3—Zn1—O1—C1	−68.3 (2)	C1—C2—C4—C3^i^	179.4 (2)
O5—Zn1—O1—C1	156.34 (17)	Zn1—O3—C5—O4	6.8 (4)
N1—Zn1—O1—C1	53.3 (2)	Zn1—O3—C5—C6	−174.73 (17)
O1—Zn1—O3—C5	−23.0 (2)	O4—C5—C6—C7	166.5 (3)
O5—Zn1—O3—C5	110.6 (2)	O3—C5—C6—C7	−12.1 (4)
N1—Zn1—O3—C5	−147.4 (2)	O4—C5—C6—C8	−13.7 (4)
O3—Zn1—O5—C9	−72.17 (19)	O3—C5—C6—C8	167.7 (2)
O1—Zn1—O5—C9	69.27 (19)	C8—C6—C7—C8^ii^	−0.1 (5)
N1—Zn1—O5—C9	−178.56 (18)	C5—C6—C7—C8^ii^	179.7 (3)
O3—Zn1—N1—C14	−168.38 (18)	C7—C6—C8—C7^ii^	0.1 (5)
O1—Zn1—N1—C14	57.3 (2)	C5—C6—C8—C7^ii^	−179.7 (3)
O5—Zn1—N1—C14	−53.78 (19)	Zn1—O5—C9—O6	5.0 (4)
O3—Zn1—N1—C13	−45.2 (2)	Zn1—O5—C9—C10	−174.5 (2)
O1—Zn1—N1—C13	−179.48 (17)	O6—C9—C10—C12	178.8 (3)
O5—Zn1—N1—C13	69.45 (19)	O5—C9—C10—C12	−1.7 (4)
Zn1—O1—C1—O2	2.5 (3)	O6—C9—C10—C11	−3.2 (5)
Zn1—O1—C1—C2	−176.58 (18)	O5—C9—C10—C11	176.3 (3)
O2—C1—C2—C4	3.4 (4)	C12—C10—C11—C11^iii^	−1.4 (3)
O1—C1—C2—C4	−177.5 (2)	C9—C10—C11—C11^iii^	−179.46 (18)
O2—C1—C2—C3	−178.0 (3)	C11—C10—C12—C12^iii^	1.4 (4)
O1—C1—C2—C3	1.1 (4)	C9—C10—C12—C12^iii^	179.40 (19)
C4—C2—C3—C4^i^	−0.8 (4)	C18—N3—C19—O7	−122.2 (3)
C1—C2—C3—C4^i^	−179.4 (2)	C17—N3—C19—O7	65.0 (3)
C3—C2—C4—C3^i^	0.8 (4)		

Symmetry codes: (i) −*x*+1, −*y*+1, −*z*+2; (ii) −*x*+2, −*y*+1, −*z*+2; (iii) *x*, −*y*+3/2, *z*.

### Hydrogen-bond geometry (Å, °)

**Table:**

*D*—H···*A*	*D*—H	H···*A*	*D*···*A*	*D*—H···*A*
N1—H1A···O4^iv^	0.91	2.27	3.109 (3)	152
N1—H1A···O2	0.91	2.54	3.040 (3)	115
N2—H2A···O4^v^	0.91	1.93	2.770 (3)	153
N2—H2B···O15	0.89	2.64	3.241 (7)	127
O8—H8X···O12^vi^	0.85	2.09	2.519 (9)	111
O8—H8X···N3^v^	0.85	2.59	3.329 (5)	147
O9—H9X···O9^iii^	0.85	1.61	2.063 (11)	110
O10—H10X···O7	0.85	1.95	2.524 (7)	124
O11—H11Y···O9	0.85	2.47	2.927 (9)	115
O11—H11X···O15^vi^	0.85	1.73	2.552 (10)	161
O12—H12X···O8^vii^	0.85	2.04	2.519 (9)	115
O13—H13F···O13^iii^	0.85	1.77	2.460 (14)	137
O13—H13F···O14	0.85	2.08	2.650 (10)	124
O15—H15X···O11^vii^	0.85	2.13	2.552 (10)	110
O16—H16X···O12	0.85	2.22	2.709 (9)	116
O16—H16X···O13	0.85	2.37	3.131 (9)	150
O16—H16Y···O10	0.85	2.62	3.079 (9)	116

Symmetry codes: (iv) −*x*+3/2, −*y*+1, *z*−1/2; (v) *x*, *y*, *z*−1; (vi) *x*+1/2, *y*, −*z*+1/2; (iii) *x*, −*y*+3/2, *z*; (vii) *x*−1/2, *y*, −*z*+1/2.

## References

[bb2] Bruker (2004). *SADABS*, *SMART* and *SAINT* Bruker AXS Inc., Madison, Wisconsin, USA.

[bb3] Clausen, H. F., Poulsen, R. D., Bond, A. D., Chevallier, M. A. S. & Iversen, B. B. (2005). *J. Solid State Chem.* **178**, 3342–3351.

[bb4] Dai, J. C., Wu, X. T., Hu, S. M., Fu, Z. Y., Zhang, J. J., Du, W. X., Zhang, H. H. & Sun, R. Q. (2004). *Eur. J. Inorg. Chem* pp. 2096–2106.

[bb5] Dybtsev, D. N., Chun, H., Yoon, S. H., Kim, D. & Kim, K. (2004). *J. Am. Chem. Soc* **126**, 32–33. 10.1021/ja038678c 14709045

[bb6] Go, Y. B., Wang, X. Q. & Jacobson, A. J. (2007). *Inorg. Chem.* **46**, 6594–6600. 10.1021/ic700693f 17625840

[bb7] Guo, H. D., Guo, X. M., Batten, S. R., Song, J. F., Song, S. Y., Dang, S., Zheng, G. L., Tang, J. K. & Zhang, H. J. (2009). *Cryst. Growth Des.* **9**, 1394–1401.

[bb8] Hawxwell, S. M., Adams, H. & Brammer, L. (2006). *Acta Cryst.* B**62**, 808–814. 10.1107/S0108768106033283 16983162

[bb9] He, X., Lu, C. Z., Yuan, D. Q., Chen, L. J., Zhang, Q. Z. & Wu, C. D. (2005). *Eur. J. Inorg. Chem* pp. 4598–4606.

[bb10] Kitagawa, S., Kitaura, R. & Noro, S. (2004). *Angew. Chem. Int. Ed* **43**, 2334–2375. 10.1002/anie.200300610 15114565

[bb11] Robin, A. Y. & Fromm, K. M. (2006). *Coord. Chem. Rev* **250**, 2127–2157.

[bb12] Rowsell, J. L. C., Millward, A. R., Park, K. S. & Yaghi, O. M. (2004). *J. Am. Chem. Soc* **126**, 5666–5667. 10.1021/ja049408c 15125649

[bb13] Rowsell, J. L. C. & Yaghi, O. M. (2004). *Micropor. Mesopor. Mater.* **73**, 3–14.

[bb14] Sheldrick, G. M. (2008). *Acta Cryst.* A**64**, 112–122. 10.1107/S0108767307043930 18156677

[bb15] Suh, M. P., Cheon, Y. E. & Lee, E. Y. (2008). *Coord. Chem. Rev.* **252**, 1007–1026.

[bb16] Tranchemontagne, D. J., Hunt, J. R. & Yaghi, O. M. (2008). *Tetrahedron*, **64**, 8553–8557.

[bb17] Wang, X. W., Chen, J. Z. & Liu, J. H. (2007). *Cryst. Growth Des.* **7**, 1227–1229.

[bb18] Wang, F. K., Yang, S. Y., Huang, R. B., Zheng, L. S. & Batten, S. R. (2008). *CrystEngComm*, **10**, 1211–1215.

[bb19] Wu, C. D., Hu, A., Zhang, L. & Lin, W. (2005). *J. Am. Chem. Soc* **127**, 8940–8941. 10.1021/ja052431t 15969557

[bb20] Zhu, L.-N., Gao, S. & Ng, S. W. (2007). *Acta Cryst.* E**63**, m2987–m2988.

